# A Weighted NBTI/HCD Coupling Model in Full VG/VD Bias Space with Applications to SRAM Aging Simulation

**DOI:** 10.3390/mi17010101

**Published:** 2026-01-12

**Authors:** Zhen Chai, Zhenyu Wu

**Affiliations:** Faculty of Integrated Circuit, Xidian University, Xi’an 710126, China; chaiz7688@163.com

**Keywords:** physical mechanism of failure, weighting factor, negative bias temperature instability (NBTI), hot carrier degradation (HCD)

## Abstract

In this paper, a coupled negative bias temperature instability (NBTI)/hot carrier degradation (HCD) failure model is proposed on the 2-D voltage plane for aging simulation of SRAM circuits. According to the physical mechanism of failure, based on the reaction–diffusion and hot carrier energy-driven theory, revised degradation models of threshold voltage shift (∆Vth) for the NBTI and HCD are established, respectively, with explicit expressions for gate voltage (V_G_)/drain voltage (V_D_). An NBTI/HCD coupling model is built on the 2-D {V_G_, V_D_} voltage plane with a weighting factor in the form of V_G_ and V_D_ power law. The model also takes into account the AC effect and long-term saturation behavior. The predicted ∆Vth under various stress conditions shows an average relative error of 11.6% with experimental data across the entire bias space. SRAM circuit simulation shows that the read static noise margin (RSNM) and write static noise margin (WSNM) have a maximum absolute error of 4.2% and 3.1%, respectively. This research provides a valuable reference for the reliability simulation of nanoscale integrated circuits.

## 1. Introduction

As technology develops, reliability concerns have become more significant [1]. Under real operating conditions in circuits, transistors often experience negative bias temperature instability (NBTI), hot carrier degradation (HCD), and mixed-mode aging, which result in changes to circuit delay and a reduction in design margins [2,3,4,5]. Precise predictions of aging-related temporal shifts and effective EDA solutions are essential for designing with reliability in mind [6].

Extensive research has been dedicated to independently elucidating the degradation mechanisms of NBTI and HCD. NBTI modeling is typically categorized into reaction–diffusion (R-D) [7] and trapping–detrapping (T-D) [8] models. The R-D model effectively captures the power-law dependence of ∆Vth, whereas the T-D framework explains charge-trapping dynamics through first-principles-based formulations. A recent advancement in the defect-centric model (DCM) uses statistical methods to analyze the random aging phenomenon in NBTI [9]. Some investigations have developed the four-state nonradiative multiphonon (NMP) model, which accurately captures charge T-D dynamics [10]. For HCD, predictive models include the lucky electron model [11,12] and the energy-driven model [13]. Many new studies have been conducted on HCD as technology has advanced. Under extended stress, the time-dynamic shape returns to a power-law trend with soft saturation [14,15]. The carrier energy distribution function (EDF) is linked to the mechanism of defect generation, and the ab initio quantum chemistry method is employed to reveal and describe the intricate physics underlying HCD [16]. In addition, the time variability [17] and Joule heating effect [18] cannot be ignored when studying NBTI/HCD. In ultrathin oxide MOSFETs, process engineering enables suppression of hole traps (N_HT_), thereby establishing interface traps (N_IT_) as the dominant degradation pathway. R-D formulations offer computationally efficient long-term predictions by approximating N_IT_ generation as a diffusion-limited process. This simplification obviates the need for exhaustive experimental calibration while maintaining predictive accuracy across extended stress durations.

However, real circuits are often affected by both NBTI and HCD simultaneously, making the modeling of their coupling effects crucial. Devices under real circuit operation are exposed to varying V_G_ and V_D_ (from 0 to VDD), and simulation extrapolation of independent degradation mechanisms yields inaccurate lifetime predictions. If NBTI and HCD are independent, experiments with alternating them stress should yield the same degree of degradation, but there are differences in the experimental results [19]. An acceleration factor method is used to sum the failure rates of various known failure mechanisms, combining their observed effects under high/low temperature and high-/low-voltage stress [20]. There is no interaction between high-energy and low-energy HCD, off-state stresses, and other modes, whereas there is a complete interaction between BTI stresses and the low-energy HCD modes [21]. Considering the influence of process variability, the contribution of N_IT_ and oxide traps (N_OT_) in BTI and HCD has been analyzed, and the entire framework has been modeled as the superposition of interdependent aging effects [22]. Recent studies have determined the contribution of different types of traps using advanced characterization techniques, identifying three types of traps from HCD experiments to model the coupling effect [23]. If a single failure model is used to predict ∆Vth in the mixed-mode region, it would lead to overly optimistic predictions [24]. In addition, the strongly correlated weighting coefficients used to superimpose the NBTI and HCD may lead to questionable estimation [25]. Two fundamental limitations persist in reliability modeling. First, existing frameworks predominantly treat NBTI and HCD as mutually independent phenomena, thereby neglecting the nonlinear interplay between defect generation and evolution under the concurrent, time-varying V_G_ and V_D_. Second, the voltage-coupling effect remains physically uncharacterized; the empirical expressions currently in use embed V_G_ and V_D_ as implicit variables, precluding the quantitative assessment of defect evolution path dependence across distinct bias spaces.

This paper first establishes the NBTI/HCD model as a function of bias voltage and builds the NBTI/HCD coupling model on the 2-D {V_G_, V_D_} voltage plane through voltage weights that consider additional effects. The coupling model is equivalent to a voltage-controlled voltage source (VCVS) injected into the gate of a critical MOSFET to simulate the effect of voltage stresses, enabling the prediction of degradation in SRAM-related circuit parameters.

## 2. Model

We focus on modeling the effects of N_IT_. N_OT_ is usually associated with high-field stresses (e.g., time-dependent dielectric breakdown (TDDB)). Typical stress conditions in NBTI and HCD have a relatively weak contribution from N_OT_. These charges can be released through tunneling or annealing, both of which have a lesser impact on long-term degradation. N_HT_ is mainly found in P-type devices and is associated with hole trapping in NBTI. However, its effect is often considered transient, and irreversible degradation is mainly caused by the accumulation of N_IT_. Of course, the contributions of N_HT_ and N_OT_ can also be considered similarly to N_IT_ [26].

We assume that the concentration of interfacial traps (ΔN_IT_) is proportional to the hole concentration (P) in the antipattern layer [27], where ΔV_th_ = qΔN_IT_/Cox and P = Cox (V_G_ − Vth). To facilitate the subsequent coupling model construction, the separate voltage-dependent models of NBTI and HCD need to be corrected first.

### 2.1. Revision of Independent Mechanism Modeling

The proposed modified NBTI model is

(1) $$\Delta V_{\text{th-NBTI}}\;\sim \;V_{G}^{\alpha }\;\exp\;(\beta \cdot V_{G})\;t^{n_{\mathrm{NBTI}}}{\left(\frac{\gamma \cdot \mathrm{PDC}}{1+\sqrt{\delta \cdot \left(1-\mathrm{PDC}\right)}}\right)}^{\varepsilon }$$

where *n*_NBTI_ is the time index, PDC is the pulse duty cycle, and *α*, *β*, *γ*, *δ*, and *ε* are the fitting parameters.

N_IT_ is functionally related to the fracture rate of the Si-H bond (k_F_). Some works model k_F_ as an exponential function of the perpendicular electric field in the gate oxide [27,28]. We present its relation to the oxide electric field (E_ox_) in the exponential form of V_G_, where the power-law form of V_G_ represents the relationship between ΔN_IT_ and P. The R-D model for N_IT_ is widely accepted by experimental validation, which illustrates the power-law time dependence of NBTI degradation and *n*_NBTI_ = 1/6 [29,30]. Based on the DC model, PDC is modeled as a scaling factor for AC degradation prediction [31]. In the frequency range of Hz–GHz, NBTI is independent of the input signal frequency [32,33].

The proposed modified HCD model is

(2) $$\Delta V_{\text{th-HCD}}\;\sim \;V_{G}^{\theta }\;\exp\left(\lambda \cdot V_{D}\right)\cdot \left(1-\exp\left(-\rho \cdot \;t^{n_{\mathrm{HCD}}}\right)\right)\;\cdot \;f^{\sigma }$$

where *n*_HCD_ is the time index, *f* is the AC frequency, and *θ*, *λ*, *ρ*, and *σ* are the fitting parameters.

The recovery effect is neglected since the trap is generated only near the drain of the transistor [34]. The power-law form of V_G_ characterizes the effect of carrier concentration, and the exponential form of V_D_ characterizes the effect of carrier energy. A larger leakage voltage results in a stronger horizontal electric field in the channel, which increases the carrier energy and the probability of N_IT_. The obtained generation rate of N_IT_ is a power law with respect to time, where *n*_HCD_ = 0.5 [12,35]. The time shift of the Si-H bond breaking position and the dispersion of the Si-O bond energy are used to explain the degenerate saturation behavior of HCD [14,36]. From the energy point of view, the frequency is modeled as a simple power-law form [37,38]. The delay degradation of the circuit is approximately the same for different duty cycles at a fixed frequency; HCD is independent of the input signal duty cycle [39].

### 2.2. Coupling Model

Based on the voltage forms derived from the independent NBTI and HCD models, we propose the coupling weight V_G_^x^ · V_D_^y^ is composed of the power-law form V_G_ and V_D_ to capture the NBTI/HCD interaction, where *x* and y are fitting parameters. We combine the power law of V_G_ and V_D_, and the final simplified coupling model is

(3) ΔVth-total = ANBTI · VGα expβ · VG · VDy1 · tnNBTI·γ·PDC1+δ·1−PDCε+ AHCD · VGθ expλ · VD·VDy2· 1−exp−ρ · tnHCD · fσ

where *A*_NBTI_ and *A*_HCD_ are fitting parameters. In NBTI experiments (e.g., time-dependent defect spectroscopy, TDDS), V_D_ is typically set to a low value, keeping the transistor in the linear region [40]. At this point, the channel carrier concentration is uniform, and the electric field is predominantly perpendicular to the gate, with a relatively weak lateral electric field. In contrast, HCD occurs at high V_D_, where the transistor enters the saturation region, channel pinch-off points appear, and the lateral electric field intensifies. High-energy carriers (hot electrons or holes) generate interface states or oxide defects through collision ionization. High V_D_ enhances the lateral electric field, affecting defect dynamics by shifting the defect energy level [41]. This alters the capture/emission energy barrier, thereby influencing capture/emission time. High-stress regions cause rapid trap capture, leading to significant threshold voltage degradation [16]. Therefore, the V_D_ term not only reflects the influence on the NMP process in NBTI but also demonstrates its impact on energy in HCD. HCD affects the channel carrier EDF via the V_D_, thereby altering the nonradiative multiphonon transition rates that govern NBTI. The channel carrier concentration is directly influenced by NBTI through V_G_, and it then impacts the HCD process through the energy-driven mechanism. When V_D_ is fixed and V_G_ increases, or V_G_ is fixed and V_D_ increases, both NBTI and HCD will increase.

**Figure 1:**
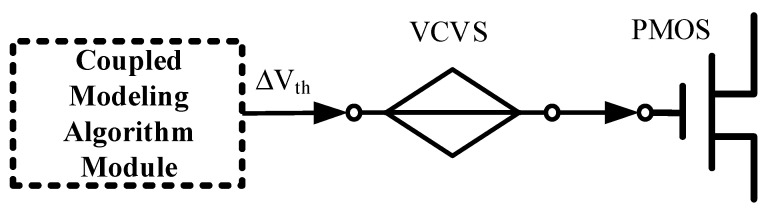
Schematic of circuit aging simulation based on coupling failure injection equivalent voltage source model.

Figure 1 shows a schematic of the circuit aging simulation based on the coupling failure injection equivalent voltage source model. This paper uses the commercial 28 nm process library at standard temperature. The PMOS transistor aspect ratio is set to 100 nm/30 nm, and the NMOS transistor aspect ratio is set to 200 nm/30 nm, where VDD = 1.8 V. The coupling model is written as an algorithmic module using Verilog-A, and the VCVS is added to the gate of the critical PMOS transistor to simulate the NBTI and HCD failure stresses applied at the relevant times.

## 3. Results and Discussion

**Figure 2:**
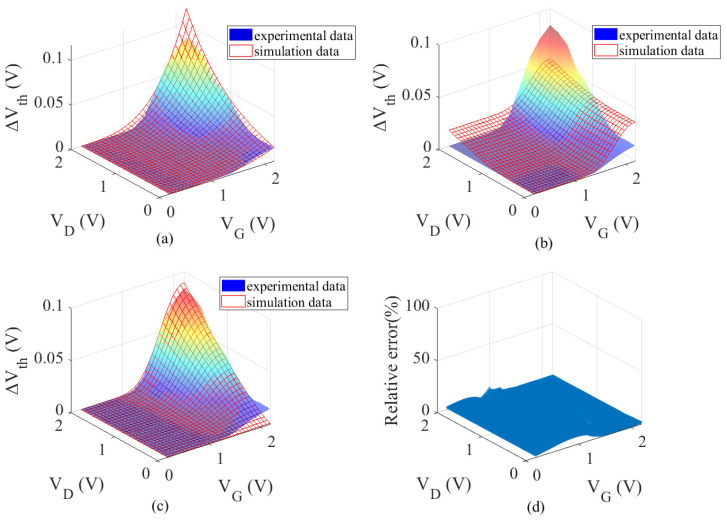
Results of fitting analysis of ∆Vth on the 2-D {V_G_, V_D_} voltage plane [42]. (**a**) NBTI model, (**b**) HCD model, (**c**) NBTI and HCD coupling model, (**d**) relative error.

Figure 2 gives the fitting results of ΔV_th_ on the 2-D {V_G_, V_D_} voltage plane. Figure 2a shows the NBTI model fitting results. In the high-voltage combination region, the maximum relative error is 23.306%, while the minimum is recorded at 0.017%. In the low-voltage combination region, the simulation data is marginally lower than the experimental data [42] and is appropriate when V_G_ = V_D_ = 2 V. Figure 2b shows the HCD model fitting result, with a maximum relative error of 39.106% and a minimum of 15.595% in the high-voltage combination region. The predicted values are higher than the experimental data [42] in the low-voltage combination region (V_G_ > 1.5 V, V_D_ < 1 V or V_G_ < 1.5 V, V_D_ > 1 V). This is due to not accounting for the contribution of NBTI under high-stress conditions. As illustrated in Figure 2c, the coupling model demonstrates a substantial discrepancy between the simulated and experimental data [42] in the low-voltage region (V_G_ < 1 V, V_D_ < 1 V), attributable to Vth degradation. Due to the smaller ΔV_th_ in the low-voltage region, minor measurement errors or model deviations are amplified, leading to poor fitting results. Therefore, we focus on the decay trend of the threshold voltage. The coupling model’s entire region has a mean square error that is normalized to 0.0641. In the region where V_G_ > 1.5 V and V_D_ > 1.5 V, the maximum value is 23.3%, and the minimum is 0.017%. The coupling weight mechanism plays a crucial compensatory and corrective role. In the range of 0–2 V across the entire bias space, Figure 2d is a plot of the effect of the fitted relative error, which is within 15% of the experimental data.

**Figure 3:**
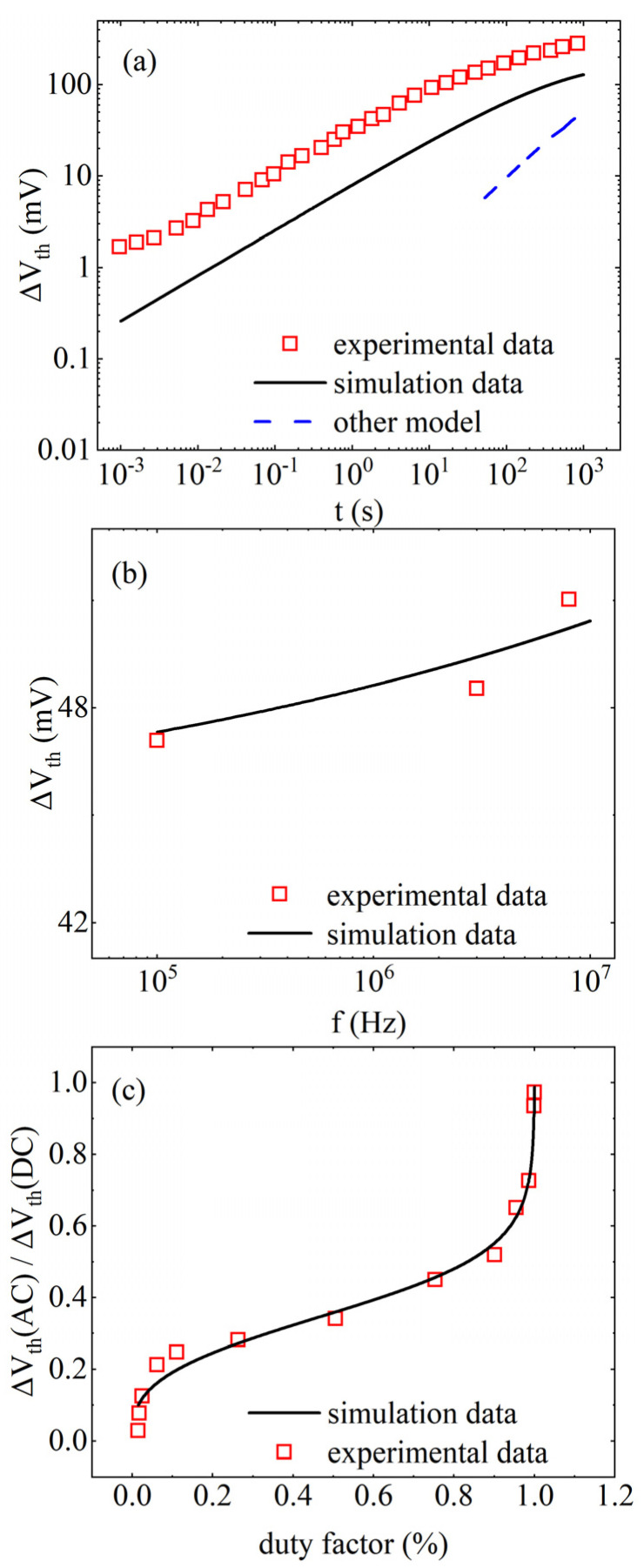
Variation curves of ∆Vth with model parameters. (**a**) HCD long-term saturation [43,44], (**b**) HCD frequency [45], (**c**) NBTI duty cycle effect [46].

Figure 3 shows the variation curves of ∆Vth with model parameters. Figure 3a shows validation of the saturation effect of the HCD, where *A*_HCD_ = 3.245, *θ* = 0.436, *λ* = 3.518, and *ρ* = 0.050. It has a similar trend of variation as the experimental data [43]. Only a single degradation mechanism of HCD is considered here, which is somewhat different in magnitude from the coupled experimental data. When the long-term saturation of HCD is not considered, the prediction after 100 s continues as a straight line [44]. The results in Figure 3b are consistent with the experimental data [45] on the HCD frequency effect. Figure 3c shows that the model is able to predict experimental data [46] for smaller and larger ranges of duty cycle, such as ranges with a duty cycle between 0.2 and 0.8.

**Figure 4:**
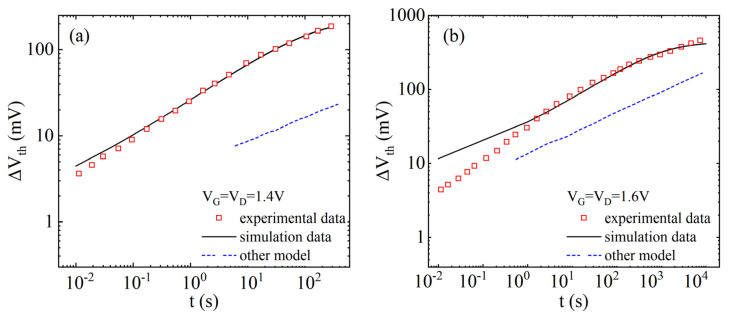
Model validation and comparison of threshold voltage degradation (∆Vth) curves with time, considering NBTI/HCD coupling failure [43,47]. (**a**) V_G_ = V_D_ = 1.4 V, (**b**) V_G_ = V_D_ = 1.6 V.

Figure 4 shows the failure of NBTI/HCD coupling in the model curves of ∆Vth with time for different bias voltages, and the relevant fitting parameters are shown in Table 1. It shows that ∆Vth has a power law that depends on time during the initial stage, while a trend of degradation saturation appears in the later stage. The failure models proposed in other works [47] cannot predict the long-term saturation behavior and differ greatly from the experimental data [43]. With the increase in stress time, the prediction error gradually decreases, indicating that the proposed coupling model has a relatively accurate long-term prediction effect.

**Figure 5:**
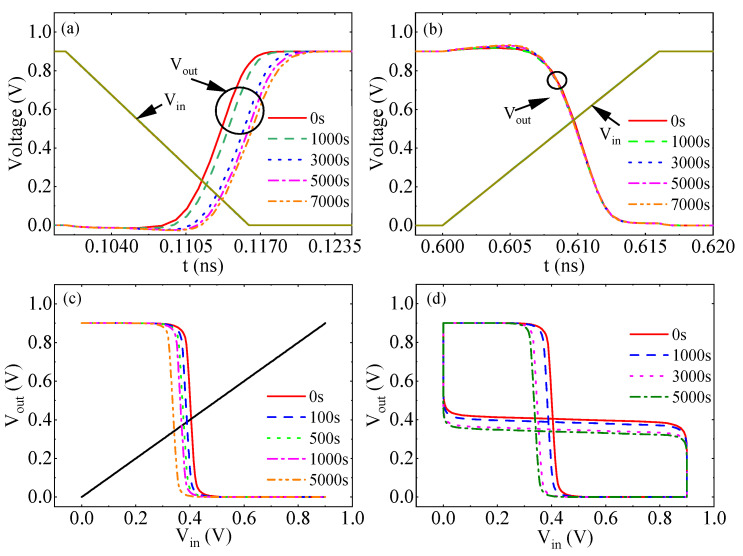
Aging simulation results of inverter NBTI/HCD coupling failure performance. (**a**) Rise delay, (**b**) fall delay, (**c**) transmission characteristics, (**d**) SNM.

Figure 5 shows the simulated performance parameters for inverter NBTI/HCD coupling failure. With increasing stress voltage time, the rise delay (Figure 5a) is degraded from 5.316 ps to 8.019 ps between 0 s and 7000 s. The fall delay (Figure 5b) is basically unchanged and degraded from 2.082 ps to 2.145 ps for the same degradation time. This is due to the fact that the rise delay is affected by the PMOS, while the fall delay is dominated by NMOS. As the severity of the PMOS transistor ∆Vth increases, the rise delay of the inverter also increases. This is consistent with the experimental results. The coupling failure leads to a leftward shift of the DC voltage transfer characteristic curve and a decrease in the switching Vth, as shown in Figure 5c. When ∆Vth shifts caused by subjecting the PMOS transistor to stress for 1000 s, 3000 s, and 5000 s are applied to the PMOS transistor, the SNMs at the four times are 0.524 V, 0.512 V, 0.47 V, and 0.457 V, respectively. As the stress time decreases from 0 s to 5000 s, the SNM of the inverter decreases from 0.524 V to 0.457 V, as shown in Figure 5d. Coupling failure leads to the weakening of the driving capability of the PMOS transistor.

**Figure 6:**
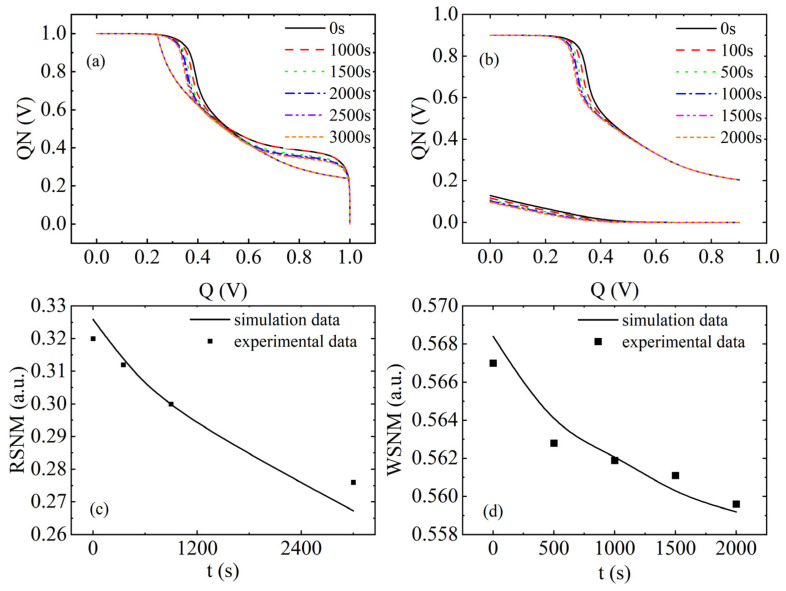
Simulation results of NBTI/HCD coupled aging and failure of a 6T-SRAM cell. (**a**) RSNM butterfly curve, (**b**) WSNM butterfly curve, (**c**) comparison of RSNM simulation results with experimental data [48], (**d**) comparison of WSNM simulation results with experimental data [48].

Figure 6 shows the simulated performance parameters associated with NBTI/HCD coupling failure in 6T-SRAM. Figure 6a plots the butterfly curves of RSNM at different times. As the stress time changes from 0 s to 3000 s, the RSNM simulation data from 0.325 V to 0.267 V demonstrates an 18% degradation, and the experimental data [48] demonstrates a 13.8% degradation. Figure 6b plots the butterfly curve of the WSNM at different times. As the stress time changes from 0 s to 2000 s, the WSNM simulation data from 0.568 V to 0.559 V demonstrates a 16.2% degradation, and the experimental data [48] demonstrates a 13.1% degradation. It can be seen that ∆Vth deepens with increasing stress time, and Vth of the PMOS transistor increases, thereby gradually reducing the stability of the circuit.

**Figure 7:**
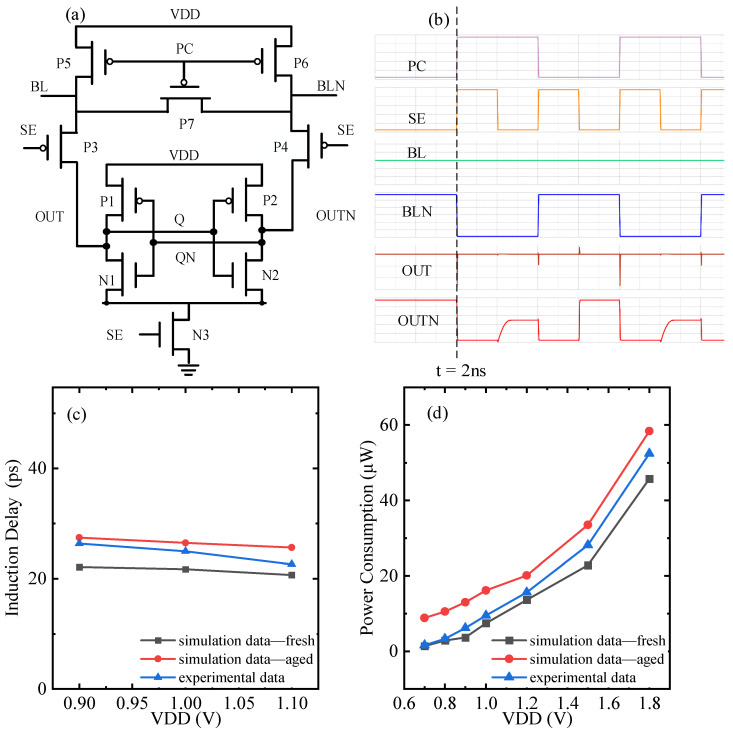
Sensitive amplifier circuit simulation results under NBTI/HCD coupling aging and failure. (**a**) Schematic, (**b**) timing simulation schematic, (**c**) sense delay degradation simulation with comparative verification at different VDDs [49], (**d**) power consumption degradation simulation with comparative verification at different VDDs [49].

Figure 7 shows the simulation results for the degradation parameters and failure performance of the sensitive amplifier under NBTI/HCD coupling. In order to investigate the effect of coupling failure on sensing delay at low power consumption, different VDD simulations are scanned, where the BTI effect is considered in the experimental data. At the 2 ns moment in Figure 7b, SE changes from 0 to 1, P3 and P4 turn off, N3 conducts, and the inverter pair begins to amplify the voltage difference on the bit line. In order to calculate the value of the sensing delay, the time difference between the moment of swing stabilization and t = 2 ns is determined. For 100 s stress time, Figure 7c shows that the sensing delay decreases with increasing VDD. The coupling failure leads to an increase in sensing delay at the same VDD. The simulation data differs from the experimental data [49] by 0.75% at VDD = 0.9 V. This is due to the fact that the N1 transistor Vth is degraded under the effect of coupling failure, so the QN voltage takes longer to drop to (VDD-Vth_-N1_). The time for the bit line to amplify to the full swing is increased, and the read speed is reduced. Figure 7d shows that the degradation of dynamic power consumption increases with increasing VDD for 800 s stress time. The bit line is connected to a large number of memory cells, making the load capacitance and load resistance increase significantly. When the sensitive amplifier performs a read operation, turning on only one row of memory cells will not only greatly prolong the data reading time but also consume a lot of power during the charging and discharging process. In low-power operating scenarios, the significant impact of coupling failure on sensing delay and power consumption must also be taken into consideration.

## 4. Conclusions

To explicitly account for the concurrent influence of the interface electric field (controlled by V_G_) and the hot carrier injection energy (driven by V_D_) on defect generation, the cross-coupling term V_G_/V_D_ is incorporated into both the R-D and hot carrier energy-driven frameworks. This augmentation yields a unified NBTI/HCD model whose analytical formulation explicitly contains V_G_ and V_D_, thereby enabling accurate prediction of defect density under arbitrary bias conditions. The final coupling model is constructed by using the voltage weight factor on the 2-D {V_G_, V_D_} voltage plane.

By calibrating with the experimental data of the 28 nm process, the proposed coupling model predicts the magnitude of degradation well under different stress conditions. The results of the circuit-level simulation method show that the errors between the simulation data and the experimental data for different metrics are within reasonable limits, which could be used to predict the performance degradation of nanoscale integrated circuits in real operating environments.

## Figures and Tables

**Table 1:** List of coupling failure model parameters for V_G_ = V_D_ = 1.4 V and 1.6 V.

V_G_ = V_D_ = 1.4 V			
*A*_NBTI_	0.979	*A*_HCD_	2.448
*α*	0.723	*θ*	5.967
*β*	1.645	*λ*	1.710
*γ*	1.140	*ρ*	0.114
*δ*	263.495	*n*_NBTI_	0.16
*ε*	0.333	*n*_HCD_	0.5
*y*1	4.129	*y*2	63.316
V_G_ = V_D_ = 1.6 V			
*A*_NBTI_	1.245	*A*_HCD_	5.052
*α*	1.096	*θ*	7.221
*β*	2.068	*λ*	0.483
*γ*	1.140	*ρ*	0.048
*δ*	263.495	*n*_NBTI_	0.16
*ε*	0.333	*n*_HCD_	0.5
*y*1	12.632	*y*2	87.137

## Data Availability

Dataset available on request from the authors.

## Abbreviations

The following abbreviations are used in this manuscript:

NBTI	Negative Bias Temperature Instability
HCD	Hot Carrier Degradation
RSNM	Read Static Noise Margin
WSNM	Write Static Noise Margin
R-D	Reaction–Diffusion
T-D	Trapping–Detrapping
DCM	Defect-Centric Model
NMP	Nonradiative Multiphonon
EDF	Energy Distribution Function
TDDB	Time-Dependent Dielectric Breakdown
VCVS	Voltage-Controlled Voltage Source

## References

[1] Sharma U., Parihar N., Mahapatra S. (2019). Modeling of HCD Kinetics for Full VG/VD Span in the Presence of NBTI, Electron Trapping, and Self Heating in RMG SiGe p-FinFETs. IEEE Trans. Electron Devices.

[2] Mahapatra S., Parihar N. (2018). A review of NBTI mechanisms and models. Microelectron. Reliab..

[3] Mahapatra S., Sharma U. (2020). A Review of Hot Carrier Degradation in n-Channel MOSFETs—Part I: Physical Mechanism. IEEE Trans. Electron Devices.

[4] Cacho F., Mora P., Arfaoui W., Federspiel X., Huard V. HCI/BTI coupled model: The path for accurate and predictive reliability simulations. Proceedings of the 2014 IEEE International Reliability Physics Symposium.

[5] Sengupta D., Sapatnekar S.S. (2017). Estimating Circuit Aging Due to BTI and HCI Using Ring-Oscillator-Based Sensors. IEEE Trans. Comput. Des. Integr. Circuits Syst..

[6] Wang R., Zhang Z., Sun Z., Guo Z., Lin Y., Huang R. Cross-Layer Design for Reliability in Advanced Technology Nodes: An EDA Perspective. Proceedings of the 2022 IEEE 16th International Conference on Solid-State & Integrated Circuit Technology (ICSICT).

[7] Naphade T., Goel N., Nair P.R., Mahapatra S. Investigation of stochastic implementation of reaction diffusion (RD) models for NBTI related interface trap generation. Proceedings of the 2013 IEEE International Reliability Physics Symposium (IRPS).

[8] Wirth G.I., da Silva R., Kaczer B. (2011). Statistical Model for MOSFET Bias Temperature Instability Component Due to Charge Trapping. IEEE Trans. Electron Devices.

[9] Franco J., Kaczer B., Mukhopadhyay S., Duhan P., Weckx P., Roussel P.J., Chiarella T., Ragnarsson L.-A., Trojman L., Horiguchi N. Statistical model of the NBTI-induced threshold voltage, subthreshold swing, and transconductance degradations in advanced p-FinFETs. Proceedings of the 2016 IEEE International Electron Devices Meeting (IEDM).

[10] Zhao Y., Wang L., Wu Z., Schanovsky F., Xu X., Yang H., Yu H., Lai J., Liu D., Chuai X. A Unified Physical BTI Compact Model in Variability-Aware DTCO Flow: Device Characterization and Circuit Evaluation on Reliability of Scaling Technology Nodes. Proceedings of the 2021 Symposium on VLSI Technology.

[11] Takeda E., Suzuki N. (1983). An empirical model for device degradation due to hot-carrier injection. IEEE Electron Device Lett..

[12] Bravaix A., Guerin C., Huard V., Roy D., Roux J.M., Vincent E. Hot-Carrier acceleration factors for low power management in DC-AC stressed 40nm NMOS node at high temperature. Proceedings of the 2009 IEEE International Reliability Physics Symposium.

[13] Arfaoui W., Federspiel X., Mora P., Monsieur F., Cacho F., Roy D., Bravaix A. Energy-driven Hot-Carrier model in advanced nodes. Proceedings of the 2014 IEEE International Reliability Physics Symposium.

[14] Varghese D., Alam M.A., Weir B. A generalized, IB-independent, physical HCI lifetime projection methodology based on universality of hot-carrier degradation. Proceedings of the 2010 IEEE International Reliability Physics Symposium.

[15] Qu Y., Lin X., Li J., Cheng R., Yu X., Zheng Z., Lu J., Chen B., Zhao Y. Ultra fast (<1 ns) electrical characterization of self-heating effect and its impact on hot carrier injection in 14nm FinFETs. Proceedings of the 2017 IEEE International Electron Devices Meeting (IEDM).

[16] Jech M., Tyaginov S., Kaczer B., Franco J., Jabs D., Jungemann C., Waltl M., Grasser T. First–Principles Parameter–Free Modeling of n– and p–FET Hot–Carrier Degradation. Proceedings of the 2019 IEEE International Electron Devices Meeting (IEDM).

[17] Bury E., Chasin A., Vandemaele M., Van Beek S., Franco J., Kaczer B., Linten D. Array-Based Statistical Characterization of CMOS Degradation Modes and Modeling of the Time-Dependent Variability Induced by Different Stress Patterns in the {VG, VD} bias space. Proceedings of the 2019 IEEE International Reliability Physics Symposium (IRPS).

[18] Kumar N., Pali S., Gupta A., Singh P. (2024). Self-Heating Mapping of the Experimental Device and Its Optimization in Advance Sub-5 nm Node Junctionless Multi-Nanowire FETs. IEEE Trans. Device Mater. Reliab..

[19] Jech M., Rott G., Reisinger H., Tyaginov S., Rzepa G., Grill A., Jabs D., Jungemann C., Waltl M., Grasser T. (2020). Mixed hot-carrier/bias temperature instability degradation regimes in full {VG, VD} bias space: Implications and peculiarities. IEEE Trans. Electron Devices.

[20] Bernstein J.B., Gabbay M., Delly O. (2014). Reliability matrix solution to multiple mechanism prediction. Microelectron. Reliab..

[21] Federspiel X., Rafik M., Angot D., Cacho F., Roy D. Interaction between BTI and HCI degradation in high-k devices. Proceedings of the IEEE International Reliability Physics Symposium (IRPS).

[22] Amrouch H., van Santen V.M., Henkel J. (2017). Interdependencies of Degradation Effects and Their Impact on Computing. IEEE Des. Test.

[23] Wang R., Sun Z., Li Y., Xue Y., Wang Z., Ren P., Ji Z., Zhang L., Huang R. Advanced Compact Modeling for Transistor Aging: Trap-based Approaches and Mixed-mode Coupling. Proceedings of the 2023 7th IEEE Electron Devices Technology & Manufacturing Conference (EDTM).

[24] Sutaria K.B., Ren P., Mohanty A., Feng X., Wang R., Huang R., Cao Y. Duty cycle shift under static/dynamic aging in 28nm HK-MG technology. Proceedings of the 2015 IEEE International Reliability Physics Symposium.

[25] Yu Z., Zhang J., Wang R., Guo S., Liu C., Huang R. New insights into the hot carrier degradation (HCD) in FinFET: New observations, unified compact model, and impacts on circuit reliability. Proceedings of the 2017 IEEE International Electron Devices Meeting (IEDM).

[26] Samadder T., Choudhury N., Kumar S., Kochar D., Parihar N., Mahapatra S. (2021). A Physical Model for Bulk Gate Insulator Trap Generation During Bias- Temperature Stress in Differently Processed p-Channel FETs. IEEE Trans. Electron Devices.

[27] Wang W., Reddy V., Krishnan A.T., Vattikonda R., Krishnan S., Cao Y. (2007). Compact modeling and simulation of circuit reliability for 65-nm CMOS technology. IEEE Trans. Device Mater. Reliab..

[28] Alam M.A., Mahapatra S. (2005). A comprehensive model of PMOS NBTI degradation. Microelectron. Reliab..

[29] Chen C.L., Lin Y.M., Wang C., Wu K. A new finding on NBTI lifetime model and an investigation on NBTI degradation characteristic for 1.2nm ultra thin oxide [MOSFETs]. Proceedings of the 2005 IEEE International Reliability Physics Symposium, 2005. Proceedings. 43rd Annual..

[30] Mahapatra S., Ahmed K., Varghese D., Islam A.E., Gupta G., Madhav L., Saha D., Alam M.A. On the Physical Mechanism of NBTI in Silicon Oxynitride p-MOSFETs: Can Differences in Insulator Processing Conditions Resolve the Interface Trap Generation versus Hole Trapping Controversy?. Proceedings of the 2007 IEEE International Reliability Physics Symposium Proceedings. 45th Annual.

[31] Liu X. (2018). Transistor/Gate Level Reliability Modeling. Ph.D. Dissertation.

[32] Chen G., Li M.F., Ang C.H., Zheng J.Z., Kwong D.L. (2002). Dynamic NBTI of p-MOS transistors and its impact on MOSFET scaling. IEEE Electron Device Lett..

[33] Grasser T., Kaczer B., Goes W. An energy-level perspective of bias temperature instability. Proceedings of the IEEE International Reliability Physics Symposium (IRPS).

[34] Maricau E., Gielen G. (2013). Analog IC Reliability in Nanometer CMOS.

[35] Tyaginov S.E., Starkov I., Enichlmair H., Park J.M., Jungemann C., Grasser T. (2011). Physics-based hot-carrier degradation modeling. ECS Trans..

[36] Randriamihaja Y.M., Huard V., Federspiel X., Zaka A., Palestri P., Rideau D., Roy D., Bravaix A. (2012). Microscopic scale characterization and modeling of transistor degradation under HC stress. Microelectron. Reliab..

[37] Bender E., Bernstein J.B., Bensoussan A. (2020). Reliability prediction of FinFET FPGAs by MTOL. Microelectron. Reliab..

[38] Tan S., Tahoori M., Kim T., Wang S., Sun Z., Kiamehr S. (2019). Long-Term Reliability of Nanometer VLSI Systems. Modeling. Analysis and Optimization.

[39] Naouss M., Marc F. (2016). FPGA LUT delay degradation due to HCI: Experiment and simulation results. Microelectron. Reliab..

[40] Anandkrishnan R., Bhagdikar S., Choudhury N., Rao R., Fernandez B., Chaudhury A., Parihar N., Mahapatra S. A Stochastic Modeling Framework for NBTI and TDDS in Small Area p-MOSFETs. Proceedings of the 2018 International Conference on Simulation of Semiconductor Processes and Devices (SISPAD).

[41] Bina M., Tyaginov S., Franco J., Rupp K., Wimmer Y., Osintsev D., Kaczer B., Grasser T. (2014). Predictive Hot-Carrier Modeling of n-Channel MOSFETs. IEEE Trans. Electron Devices.

[42] Sangani D., Diaz-Fortuny J., Bury E., Kaczer B., Gielen G. Assessment of Transistor Aging Models in a 28nm CMOS Technology at a Wide Range of Stress Conditions. Proceedings of the 2022 IEEE International Integrated Reliability Workshop (IIRW).

[43] Mahapatra S., Rashmi S. (2018). On the universality of hot carrier degradation: Multiple probes, various operating regimes, and different MOSFET architectures. IEEE Trans. Electron Devices.

[44] Patra D., Zhang J., Wang R., Katoozi M., Cannon E.H., Huang R., Cao Y. Compact modeling and simulation of accelerated circuit aging. Proceedings of the IEEE Custom Integrated Circuits Conference (CICC).

[45] Garba-Seybou T., Bravaix A., Federspiel X., Cacho F. (2021). Modeling hot carrier damage interaction between on and off modes for 28 nm AC RF applications. Microelectron. Reliab..

[46] Subirats A., Garros X., Cluzel J., El Husseini J., Cacho F., Federspiel X., Huard V., Rafik M., Reimbold G., Faynot O. A new gate pattern measurement for evaluating the BTI degradation in circuit conditions. Proceedings of the IEEE International Reliability Physics Symposium (IRPS).

[47] Duan M., Zhang J.F., Manut A., Ji Z., Zhang W., Asenov A., Gerrer L., Reid D., Razaidi H., Vigar D. Hot carrier aging and its variation under use-bias: Kinetics, prediction, impact on Vddand SRAM. Proceedings of the 2015 IEEE International Electron Devices Meeting (IEDM).

[48] Huard V., Parthasarathy C., Guerin C., Valentin T., Pion E., Mammasse M., Planes N., Camus L. NBTI degradation: From transistor to SRAM arrays. Proceedings of the 2008 IEEE International Reliability Physics Symposium.

[49] Agbo I., Khan S., Hamdioui S. BTI impact on SRAM sense amplifier. Proceedings of the 2013 8th IEEE Design and Test Symposium.

